# A retrospective pooled analysis of response patterns and risk factors in recurrent malignant glioma patients receiving a nitrosourea-based chemotherapy

**DOI:** 10.1186/1479-5876-10-90

**Published:** 2012-05-14

**Authors:** Alessandro Paccapelo, Ivan Lolli, Maria Grazia Fabrini, Giovanni Silvano, Beatrice Detti, Franco Perrone, Giuseppina Savio, Matteo Santoni, Erminio Bonizzoni, Tania Perrone, Silvia Scoccianti

**Affiliations:** 1 Department of Medical Oncology, Azienda Ospedaliero-Universitaria, Ospedali Riuniti Umberto I-GM Lancisi and G Salesi, Ancona, Italy; 2 Oncologia Medica, IRCCS “Saverio de Bellis”, 70013 via Turi 27, Castellana Grotte, (Ba), Italy; 3 Azienda Ospedaliero-Universitaria Pisana, Pisa, Italy; 4 SS. Annunziata Taranto Hospital, Taranto, Italy; 5 Azienda Ospedaliera Universitaria Careggi, Florence, Italy; 6 Ospedale Civico ARNAS, Palermo, Italy; 7 University of Milan, Milan, Italy; 8 Italfarmaco Sp.A., Cinisello Balsamo, (MI), Italy

**Keywords:** Fotemustine, Nitrosourea, Recurrent-glioblastoma, Stupp-regimen, Temozolomide

## Abstract

**Background:**

At recurrence the use of nitrosoureas is widely-used as a therapeutic option for glioblastoma (GBM) patients. The efficacy of fotemustine (FTM) has been demonstrated in phase II clinical trials; however, these papers report a wide range of progression-free-survival (PFS-6 m) rates, ranging from 21% to 52%. We investigated whether FTM could have a different response pattern in respect to time to adjuvant temozolomide failure, or whether specific independent risk factors could be responsible for the wide range of response rates observed.

**Methods:**

Recurrent GBM patients have been treated with fotemustine 75-100 mg/sqm at day 1, 8, 15 and after 4/5 weeks of rest with 100 mg/sqm every 21 days. Patients were stratified in 4 groups according to time to temozolomide failure: before starting (B0), during the first 6 months (B1), after more than 6 months of therapy (B2), and after a treatment-free interval (B3). Primary endpoint was PFS-6 m. A multivariable analysis was performed to identify whether gender, time after radiotherapy, second surgery and number of TMZ cycles could be independent predictors of the clinical benefit to FTM treatment.

**Results:**

163 recurrent GBM patients were included in the analysis. PFS-6 m rates for the B0, B1, B2 and B3 groups were 25%, 28%, 31.1% and 43.8%, respectively. The probability of disease control was higher in patients with a longer time after radiotherapy (p = 0.0161) and in those who had undergone a second surgery (p = 0.0306).

**Conclusions:**

FTM is confirmed as a valuable therapeutic option for patients with recurrent GBM and was active in all study patient groups. Time after the completion of radiotherapy and second surgery are independent treatment-related risk factors that were predictive of clinical benefit.

## Background

Glioblastoma (GBM) is the most common malignant primary brain tumor in adults and is associated with a poor prognosis [1]. Radiotherapy, plus concomitant and adjuvant temozolomide (TMZ), is the standard first line treatment given to GBM patients, as defined in the EORTC trial [2,3]. On recurrence of the tumor, patients have few therapeutic options – nitrosoureas, alternative TMZ schedules, and new target therapies, but prognosis remains poor [4].

Novel target therapies, including antiangiogenic drugs, are under investigation, but the role of such drugs in the treatment of GBM is still being debated [5]. Research is needed to establish the most advantageous combination regimens containing antiangiogenic or target therapies [6].

Rechallenge with TMZ is often used following GBM recurrence, but its usefulness is questionable due to conflicting data [7-9]. Recently, Perry and colleagues [10] have demonstrated that time to adjuvant temozolomide failure seems to be linked to a high-dose TMZ treatment. Continuous high-dose TMZ is used in patients who relapse after standard therapy, notably in those patients with early or late progression after standard therapy.

Fotemustine (FTM) is a third-generation chloroethylnitrosourea containing a phosphoalanine carrier group attached to the nitrosourea radical [11]. This characteristic allows FTM to cross the blood–brain barrier (BBB), as shown by experimental studies in animals [12-14]. In addition, as FTM does not significantly alter glutathione reductase activity, a more favorable pulmonary toxicity profile for this agent can be predicted compared with BCNU [15]. During a conventional treatment schedule, FTM is administered as induction therapy at a dose of 100 mg/m2/week for three consecutive weeks, followed by maintenance therapy of 100 mg/m2 every three weeks, after a 5-week rest [16]. Several Italian groups investigated the efficacy of FTM in malignant glioma patients experiencing tumor recurrence after standard TMZ treatment. In these reports, the 6-months progression-free survival (PFS-6 m) rates ranged from 21 to 52% [17-19]. Despite the homogeneous patient population in these studies (patients with the first recurrence of GBM after initiation of the Stupp regimen2), there was a high variability in the PFS-6 m rate. To justify this data, different hypotheses have been formulated. For example, the presence of patients experiencing pseudoprogression [20], as suggested recently by Silvani and colleagues [16], resulting in a different response pattern related to time of adjuvant TMZ failure, or the presence of patient- or treatment-specific risk factors in the study population.

Based on these assumptions, the aim of the present retrospective analysis was to assess whether FTM could have a different response pattern compared to the time to adjuvant TMZ failure, or whether specific independent risk factors could be responsible for the wide range of survival rates observed.

## Methods

Six Italian sites participated in this retrospective pooled analysis. The study was done in accordance with the provisions of the Declaration of Helsinki and local regulations. The study was approved by the institutional review board at each study centre.

Adult patients with recurrent or progressive, histologically-confirmed GBM who underwent surgery and the Stupp regimen (radiotherapy plus concomitant and adjuvant temozolomide) [2] were included in the analysis. Progression was documented by MRI or computed tomography (CT) scans. All patients received 1 h intravenous infusion of FTM according to the following schedule: induction phase dose of 70–100 mg/m2 on days 1, 8, 15, followed by a 4/5-week rest period, and a maintenance phase dose of 100 mg/m2 every 21 days.

Contrast-enhanced (gadolinium-DTPA 0.2 mmol/kg) MRI of the brain was uniformly adopted for tumor assessment and response evaluation. Baseline MRI examination was performed before administration of FTM, and subsequent evaluations were carried out after completion of the induction phase, every two cycles during the maintenance phase.

Glioblastoma patients were stratified into three groups according to time to TMZ failure, as proposed by Perry and colleagues10. Group B1 (early): progression while receiving adjuvant TMZ before completion of six cycles of adjuvant TMZ. Group B2 (extended): progression while receiving extended adjuvant TMZ beyond the standard six cycles, but before completion of the adjuvant treatment. Group B3 (rechallenge): progression after completion of TMZ.

We added a fourth group, B0: progression after radiotherapy completion and before initiation of adjuvant TMZ. Response was assessed clinically and radiologically using evaluated with Macdonald’s criteria [21]. Safety was evaluated during the study and all adverse events were recorded and graded according to the common terminology criteria for adverse events (CTCAE) from the National Cancer Institute, version 4.0. (http://ctep.cancer.gov/forms/CTCAEv4.pdf).

The primary endpoint of the study was evaluation of progression-free survival at six months (PFS-6 m) according to time of TMZ failure (B0, B1, B2, B3).

Secondary endpoints were evaluated in all four patient groups, plus the whole population, and were: response rate (RR), disease control (DC), overall survival at 1 year (OS-1y) and time to progression (TtoP). Responses were confirmed as complete (CR), partial (PR) and stable (SD). RR was defined as CR + PR, otherwise DC is defined as CR + PR + SD. OS was measured from the start of FTM to death for any reason, or last follow-up assessment. Disease progression (DP) was confirmed with two consecutive radiologic investigations.

A multivariable analysis of the whole population was performed to identify whether gender, time after radiotherapy, second surgery and number of TMZ cycles could be independent predictors of clinical benefit (DC) to the FTM treatment. The multivariable model was built adopting the so-called epidemiological approach, which consists of selecting covariates regardless of univariate findings or statistical elimination methods (backward, stepwise, forward etc) but depending only on their expected or potential clinical importance. Time to progression and time to death, in the whole population, was estimated by Kaplan-Meier analysis between responder and non-responder patients.

### Statistical methods

The demographic and clinical-pathological characteristics were summarized using descriptive statistics. In general, absolute and relative frequencies were employed to summarize qualitative variables, while arithmetic mean, standard deviation (SD), range, median and interquartile range were used to summarize quantitative data.

Penalized multivariable logistic regressions with gender, time from radiotherapy, second surgery, and number of TMZ cycles as covariates were performed adopting the Firth’s correction in order to adjust estimates for potential over-fitting, skewed data and multicollinearity. Results were reported as adjusted (penalized) odds ratios (ORs) with an associated 95% CI and two-tailed probability values. Statistical calculations were carried out using SAS version 9.2. A two-tailed P-value of 0.05 was used to define statistically significant results.

## Results

One hundred and sixty-three patients with a diagnosis of recurrent GBM were included in the analysis. All patients followed a Stupp regimen as first line treatment; all patients were treated with a combination of radiotherapy and TMZ, 87.7% (143/163) received adjuvant TMZ, and 12.3% (20/163) of patients experienced a recurrence immediately following the conclusion of radiotherapy and were thus evaluated as the B0 group. Patient characteristics are listed in Table 1 for the whole population according to time of adjuvant TMZ failure stratification: 12.3% (20/163), 30.7% (50/163), 27.6% (45/163), 29.4% (48/163) were divided into the B0, B1, B2, B3 groups, respectively. Most patients were male (66.9%) with a median age of 57 years (range 47–66). All patients were included in the efficacy and safety analysis.

**Table 1:** Patient characteristics according to time of TMZ failure for the entire population

**Variable**	**B0**	**B1**	**B2**	**B3**	**All**
Number of pts	20	50	45	48	163
**Age** years					
Median (range)	59.5 (50 -65)	57.5 (47 - 64)	57 (49 - 66)	57 (47 - 65)	57 (47 - 66)
**Gender** % (N)					
Male	70 (14)	64 (32)	64.4 (29)	70.8 (34)	66.9 (109)
Female	30 (6)	36 (18)	35.6 (16)	29.2 (14)	33.1 (54)
**Type of Surgery** at diagnosis % (N)					
Biopsy	0	4 (2)	2.2 (1)	2.1 (1)	2.5 (4)
Partial	55 (11)	36 (18)	44.4 (20)	39.6 (19)	41.7 (68)
Complete	45 (9)	60 (30)	53.3 (24)	58.3 (28)	55.8 (91)
**RPA** at baseline % (N)					
III	5 (1)	14 (7)	17.8 (8)	31.3 (15)	19 (31)
IV	90 (18)	68 (34)	73.3 (33)	66.7 (32)	71.8 (117)
V	5 (1)	18(9)	8.9 (4)	2 (1)	9.2 (15)
**Number of adjuvant TMZ**					
Cycles, *Mean (range)*	0	3 (2 – 4)	9 (7 – 11)	6.5 (6 – 11)	6 (2 – 8)
**Time after end of radiation**					
Months, *Mean (range)*	1 (0.7 - 1.2)	4.2 (3.1 - 5.1)	8.9 (6.7 - 11.4)	14.1 (10.7 - 25.7)	6.8 (3.9 - 12)
**Surgery** at recurrence % (N)					
No	95 (19)	88 (44)	80 (36)	75 (36)	82.8 (135)
Yes	5 (1)	12 (6)	20 (9)	25 (12)	17.2 (28)
**FTM induction dosage** (mg/m^2^)					
Median (range)	95 (80– 100)	100 (80 – 100)	100 (80 – 100)	75 (75 – 100)	100 (75 – 100)
**FTM administration number**					
Median (range)	4 (3 – 11)	4 (1 – 18)	3 (1 – 18)	5 (1 – 20)	4 (1 – 20)

FTM: fotemustine TMZ: temozolomide; RPA: recursive partitioning analysis; B0: patients failing after radiotherapy completion and before starting adjuvant TMZ; B1: patients failing during the first 6 months of adjuvant TMZ; B2: patients who failed after more than 6 months of therapy; B3: patients who experienced tumor recurrence after a treatment-free interval.

All patients received at least one dose of the study drug, with a median number of 4 administrations up to a maximum of 20 doses. During the induction phase, 100 mg/m2 of FTM was the median administered dosage (range 75–100).

Response to FTM treatment was documented in all patients (Table 2). The PFS-6 m rate, the primary endpoint of the study, was reported in 25.0% of B0, 28.0% of B1, 31.1% of B2 and 43.8% of B3.

**Table 2:** Response to FTM treatment according to time of TMZ failure for the entire population

**Variable**	**B0**	**B1**	**B2**	**B3**	**All**
Number of pts	20	50	45	48	163
**Response to FTM** % (N)					
CR	0	2 (1)	2.2 (1)	2.1 (1)	1.8 (3)
PR	15 (3)	10 (5)	15.6 (7)	16.7 (8)	14.1 (23)
SD	30 (6)	24 (12)	24.4 (11)	47.9 (23)	31.9 (52)
DP	55 (11)	64 (32)	57.8 (26)	33.3 (16)	52.1 (85)
**Disease Control** % (N)					
Yes	45 (9)	36 (18)	42.2(19)	66.7 (32)	47.8 (78)
**PFS-6 m **% (N)					
Yes	25 (5)	28 (14)	31.1 (14)	43.8 (21)	33.1 (54)
**OS-1y** % (N)					
Yes	25 (5)	24 (12)	28.9 (13)	31.3 (15)	27.6 (45)
**Time to DP after FTM**					
Days median (range)	111 (83 - 182)	91 (43 - 156)	104 (58 - 190)	139 (62 - 252)	104 (58 - 193)

CR: complete response; PR: partial response; SD: stable disease; DP: disease progression; PSF-6 m: progression free survival at 6 months; OS-1y: overall survival at 1 year; FTM: fotemustine. B0: patients failing after radiotherapy completion and before starting adjuvant TMZ; B1: patients failing during the first 6 months of adjuvant TMZ; B2: patients who failed after more than 6 months of therapy; B3: patients who experienced tumor recurrence after a treatment-free interval.

RR was reported as 15.0%, 10.0%, 15.6% and 16.7% of the B0, B1, B2 and B3 groups respectively. The DC rate ranged from 36.0% (B1) to 66.7 (B3), while OS-1y ranged from 25.0% (B0) to 31.3% (B3).

The multivariable logistic regression evaluated the impact of gender, time from radiotherapy, second surgery, and number of TMZ cycles on DC in the whole population (Table 3). The probability of DC was higher in patients with a longer time from radiotherapy (OR ± 95% CI: 1.075, 1.019-1.147; p = 0.0161) and in those who underwent a second surgery (OR ± 95% CI: 2.802, 1.152-7.334; p = 0.0306).

**Table 3:** Multivariable penalized logistic regression using Firth’s correction

**Response variable = Disease control (CR + PR + SD)**				
**Effect**	**Odds ratio**	**Lower 95% Confidence Limit**	**Upper 95% Confidence Limit**	**P-Value**
Gender - Female vs Male	0.906	0.452	1.800	0.7806
Second Surgery	2.802	1.152	7.334	**0.0306**
Time from radiotherapy	1.075	1.019	1.147	**0.0161**
Number of TMZ cycles	0.925	0.838	1.013	0.1053

Response variable is Disease control (CR + PR + SD). Odds ratios for time from radiotherapy and number of TMZ cycles show an improvement ratio (OR > 1) associated with an increase of 1 month in time from radiotherapy and the deterioration ratio (OR < 1) associated with an increase of 1 cycle in number of TMZ.

FTM administration was well tolerated and the most relevant grade 3–4 toxicity events were leucopenia (6.7%) and thrombocytopenia (9.8%), as expected. No differences in tolerability were observed between study groups (Table 4). None unexpected toxicity has been detected in the study population.

**Table 4:** Patient safety profile according to time of temozolomide failure, and for the entire population

**Variable**	**B0**	**B1**	**B2**	**B3**	**All patients**
**Number of patients**	20	50	45	48	163
**Leucopoenia**					
** G** 0	65 (13)	78 (39)	68.9 (31)	62.5 (30)	69.3 (113)
** G** 1-2	20 (4)	20 (10)	18.9 (13)	25 (12)	23.9 (39)
** G** 3-4	15 (3)	2 (1)*	2.2 (1)*	12.5 (6)	6.7 (11)
**Thrombocytopenia**					
** G** 0	65 (13)	52 (26)	62.2 (28)	66.7 (32)	60.7 (99)
** G** 1-2	25 (5)	38 (19)	35.6 (16)	16.6 (8)	19.4 (48)
** G** 3-4	10 (2)	10 (5)	2.2 (1)*	16.7 (8)	9.8 (16)
**Anemia**					
** G** 0	80 (16)	96 (48)	93.3 (42)	97.9 (47)	93.9 (153)
** G** 1-2	15 (3)	2 (1)	6.6 (3)	2.1 (1)	5 (8)
** G** 3-4	5 (1)*	2 (1)*	0	0	1.2 (2)*
**Neutropenia**					
** G** 0	90 (18)	94 (47)	100 (45)	75 (36)	89.6 (146)
** G** 1-2	5 (1)	6 (3)	0	12.5 (6)	6.1 (10)
** G** 3-4	5 (1)	0	0	12.5 (6)	4.3 (7)
**Lymphopenia**					
** G** 0	100 (20)	92 (46)	100 (45)	77.1 (37)	90.8 (148)
** G** 1-2	0	6 (3)	0	14.6 (7)	6.1 (10)
** G** 3-4	0*	2 (1)*	0*	8.4 (4)	3.1 (5)
**Hepatic**					
** G** 0	80 (16)	96 (48)	95.6 (43)	93.8 (45)	93.3 (152)
** G** 1-2	20 (4)	2 (1)	4.4 (2)	4.2 (2)	5.5 (9)
** G** 3-4	0	2 (1)	0	2.1 (1)	1.2 (2)

*No grade 4 toxicity was detected. B0: patients failing after radiotherapy completion and before starting adjuvant TMZ; B1: patients failing during the first 6 months of adjuvant TMZ; B2: patients who failed after more than 6 months of therapy; B3: patients who experienced tumor recurrence after a treatment-free interval.

## Discussion

Recurrent glioblastoma has an unfavorable prognosis, with a PFS-6 m rate ranging from 15% to 21%, and a median survival of 25 weeks. The optimal strategy for recurrent glioblastoma has not yet been defined [22]. Nitrosourea chemotherapy, carmustine and FTM, and alternative TMZ treatment regimes are often used on recurrence of the tumor [4]. Patients treated with carmustine at a dose of 80 mg/m2 on days 1 to 3 (repeated every 3 weeks for a maximum of 6 cycles) had a 13% PFS-6 m rate and a median OS of 22 weeks [23].

The clinical response to rechallenge with high-dose TMZ has been questionable, due to conflicting data [5-7], but recently the RESCUE trial [10] demonstrated that time to adjuvant TMZ failure could influence the response to TMZ treatment on tumor recurrence: PFS-6 m rates were 27.3%, 7.4%, 35.7%, while OS-1y rates were 27.3%, 14.8%, and 28.6% for patients failing during the first 6 months of adjuvant TMZ (B1), those who failed after more than 6 months of therapy (B2), and those who experienced a recurrence after a treatment-free interval (B3), respectively. Continuous high-dose TMZ seems more effective in the B1 and B3 groups [10].

FTM is a third generation nitrosourea with more favorable efficacy and safety profile compared with carmustine [24]. Recently, several phase II trials studied the use of FTM in malignant glioma patients recurring after following the Stupp regimen [17-19], but in these papers the efficacy of FTM was associated with a wide range of PFS-6 m rates, ranging from 21% to 52%, while the DC ranged from 42.5% to 62%. To justify this range, we assumed that efficacy of FTM could have a different response pattern in respect to time to adjuvant TMZ failure, or that specific independent risk factors could influence the drug activity. We retrospectively reviewed recurrent GBM patients treated with FTM and having received a Stupp regimen, and stratified them accordingly to time to TMZ failure.

In our analysis, FTM was active in all patient groups, with a higher response to the nitrosourea treatment trend in patients who experienced tumor recurrence after a treatment-free interval. For these patients, the PFS-6 m rate was 43.8%, the OS-1y rate was 31.3%, the RR was 18.8% and the DC was 66.7%. The hypothesis that a higher response to FTM treatment could be due to the presence of patients in the pseudoprogression16 phase seems not to be justified in our patient series. Patients who relapsed near the end of radiotherapy (B0 group) were those with the lowest clinical response for all study endpoints: PFS-6 m (25.0%), OS-1y (25.0%), and RR (15.0%).

**Figure 1:**
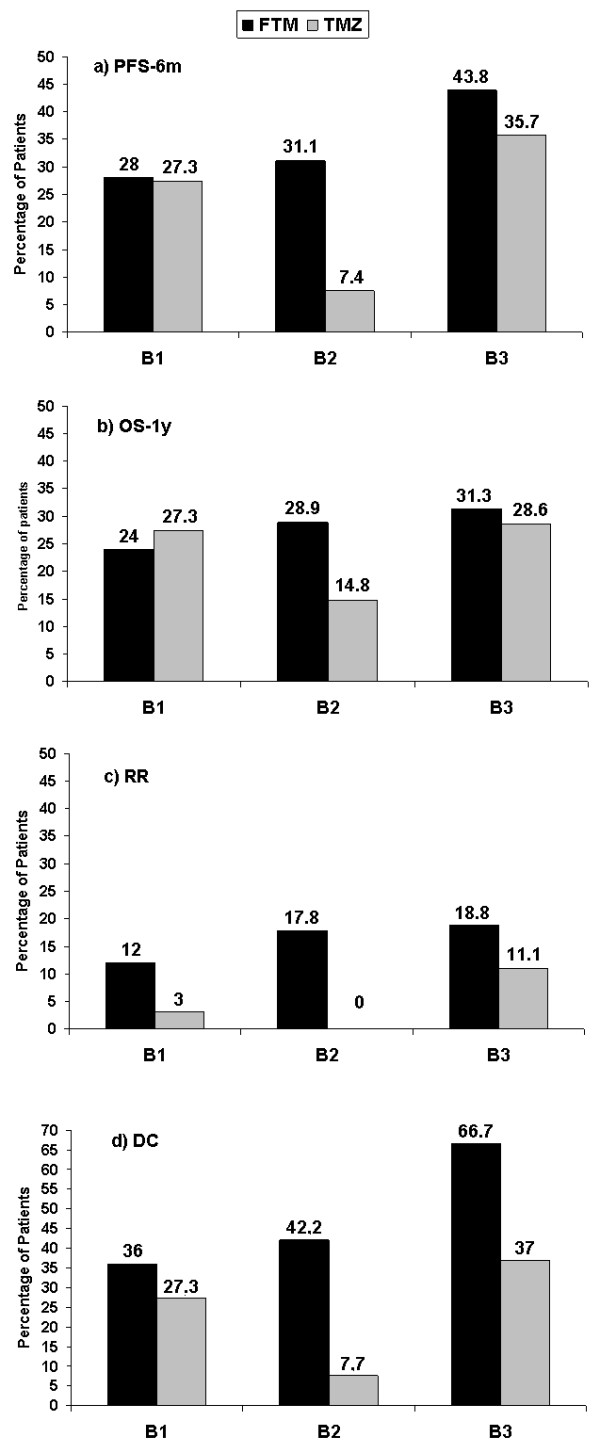
**Comparison between TMZ and FTM.** FTM: fotemustine; TMZ: temozolomide; B1: GBM patients failing during the first 6 months of adjuvant TMZ; B2: GBM patients who failed after more than 6 months of therapy; B3: GBM patients who experienced tumor recurrence after a treatment-free interval.

A comparison between our data and those reported by Perry could suggest a potentially different response pattern between recurrent GBM patients treated with FTM and those with TMZ, in respect to adjuvant TMZ failure. TMZ is active in early and late progression patients, while FTM was always active in recurrent patients: the PFS-6 m, OS-1y, RR and DC rates are reported in Figure 1. The greatest difference between the two treatments appears to be in the B2 group where FTM and TMZ have a different pattern of activity: PFS-6 m rates were 31.1% and 7.4%, OS-1y rates were 28.9% and 14.8%, RRs were 17.8% and zero, DCs were 42.2% and 7.7%.

Hypothetically, the different response pattern between patients treated with FMT and those with TMZ could be due to the different mechanism of action between the two alkylating agents. FTM, unlike TMZ, a known mono-functional DNA methylating agent, is a mono-functional/bi-functional agent containing a chloroethylating group11, acting through a mechanism largely based on DNA interstrand cross-linking [25].

The multivariable analysis investigated whether patient- (gender) or treatment- (time after radiation, number of TMZ cycles, second surgery) specific risk factors could be predictive of the FTM clinical benefit. Results of the multivariable analysis demonstrated that time after radiotherapy and second surgery are predictive of a DC in patients treated with FTM. In Figure 1 it can be appreciated how all clinical endpoints (PFS-6 m, OS-1y, RR and DC) occur at a higher rate in patients who underwent previous radiotherapy (B3), and that those with the lowest values are those in the B0 group.

In the light of these results, we could hypothesize that the conflicting data in the FTM trials could be due to the inclusion of higher risk patients. The GICNO study [17] also included patients who did not start adjuvant TMZ, while the Scoccianti [18] and Fabrini [19] studies included only patients who started adjuvant TMZ and where tumor recurrence was at least 3 months after the end of radiotherapy.

Recently the EORTC Brain Tumour Group analysed retrospectively data from 300 patients with recurrent GBM, from phase I or II trials, to evaluate patient’s age, sex, World Health Organisation (WHO) performance status (PS), presence of neurological deficits, disease history, use of steroids or anti-epileptics and disease characteristics to predict PFS and OS. This study confirms performance status but not age as a major prognostic factor for PFS and OS in recurrent GBM patients initially treated by chemoradiation with temozolomide [26]. Future prospective studies could confirm if these independent positive risk factors noticed in the EORTC trial and those of our study, could be find also in patients treated with other chemotherapy agents both for naïve and recurrent GBM patients.

Our study is biased in that the analysis was carried out retrospectively, and that the MGMT status data for the patients experiencing tumor recurrence is missing; however, we know that the methylation status of the promoter is prognostic at the time of diagnosis [27], but does not seem to be predictive of outcome at recurrence of GBM [28].

## Conclusions

FTM is confirmed as a valuable therapeutic option for patients with recurrent GBM (PFS-6 m = 33.1%), and is active in all study patient groups (B0, B1, B2, B3). In our study population gender and number of TMZ cycles are not predictive of disease control, while time after the end of radiotherapy and second surgery are independent treatment-related risk factors. These data could be useful to plan prospective and randomized studies to better define the role of chemotherapy in the combination treatment strategy of an heterogeneous cancer, like GBM.

## Competing interests

TP is an employee of Italfarmaco S.p.A. The study has been supported by an unrestricted grant by Italfarmaco S.p.A. All other authors have no financial or other conflict to declare.

## Authors’ contributions

AP, IL and SS contributed equally to this work. EB planned and reviewed the statistical analysis. All authors read and approved the final manuscript.
